# Comparative efficacy of sodium thiosulfate, bisphosphonates, and cinacalcet for the treatment of vascular calcification in patients with haemodialysis: a systematic review and network meta-analysis

**DOI:** 10.1186/s12882-024-03460-x

**Published:** 2024-01-22

**Authors:** Lei He, Yuzhe Li, Jingjing Jin, Meijuan Cheng, Yaling Bai, Jinsheng Xu

**Affiliations:** Departments of Nephrology, Hebei Key Laboratory of vascular calcification in kidney disease; Hebei Clinical Research Center for Chronic Kidney Disease, The Fourth Hospital of Hebei Medical University, 12 Jiankang Road, 050011 Shijiazhuang, China

**Keywords:** Vascular calcification, Haemodialysis, Sodium thiosulfate, Bisphosphonates, Cinacalcet

## Abstract

**Background:**

Up to now, there is no unequivocal intervention to mitigate vascular calcification (VC) in patients with hemodialysis. This network meta-analysis aimed to systematically evaluate the clinical efficacy of sodium thiosulfate, bisphosphonates, and cinacalcet in treating vascular calcification.

**Methods:**

A comprehensive study search was performed using PubMed, Web of Science, the Cochrane Library, EMBASE and China National Knowledge Internet (CNKI) to collect randomized controlled trials (RCTs) of sodium thiosulfate, bisphosphonates, and cinacalcet for vascular calcification among hemodialysis patients. Then, network meta-analysis was conducted using Stata 17.0 software.

**Results:**

In total, eleven RCTs including 1083 patients were qualified for this meta-analysis. We found that cinacalcet (SMD − 0.59; 95% CI [–0.95, -0.24]) had significant benefit on vascular calcification compared with conventional therapy, while sodium thiosulfate or bisphosphonates did not show such efficiency. Furthermore, as for ranking the efficacy assessment, cinacalcet possessed the highest surface under the cumulative ranking curve (SUCRA) value (88.5%) of lessening vascular calcification and was superior to sodium thiosulfate (50.4%) and bisphosphonates (55.4%). Thus, above results suggested that cinacalcet might be the most promising drug for vascular calcification treatment in hemodialysis patients. Mechanistically, our findings illustrated that cinacalcet reduced serum calcium (SMD − 1.20; 95% CI [–2.08, − 0.33]) and showed the tendency in maintaining the balance of intact Parathyroid Hormone (iPTH) level.

**Conclusions:**

This network meta-analysis indicated that cinacalcet appear to be more effective than sodium thiosulfate and bisphosphonates in mitigating vascular calcification through decreasing serum calcium and iPTH. And cinacalcet might be a reasonable option for hemodialysis patients with VC in clinical practice.

**Systematic Review Registration:**

[http://www.crd.york.ac.uk/PROSPERO], identifier [CRD42022379965].

**Supplementary Information:**

The online version contains supplementary material available at 10.1186/s12882-024-03460-x.

## Introduction

Vascular calcification (VC), a common complication in haemodialysis (HD) patients, is associated with stiffening of the arterial wall and disruption of blood flow and significantly increases the risk of cardiovascular-related morbidity and mortality [1–3]. Thus, improving or reversing vascular calcification is of great significance and extremely urgent. To date, numerous studies have been conducted to explore the mechanisms of vascular calcification in an attempt to identify effective therapeutic approaches. However, there is still a lack of an unequivocal intervention to consistently attenuate vascular calcification progression [4].

Recent studies have proven that VC is a complicated active pathological process involving mineral metabolism disturbance, a reduction in mineralization inhibition factors, and secondary hyperparathyroidism (SHPT) [5–7]. Sodium thiosulfate, bisphosphonates, and cinacalcet, which target the above mechanisms, are currently being used to mitigate vascular calcification in clinical practice. Studies have shown that sodium thiosulfate (STS) plays a role in maintaining the balance of bone mineral metabolism and might retard the progression of vascular calcification by chelating precipitated calcium to form soluble calcium thiosulfate [8, 9]. Adirekkiat et al. evaluated the effect of STS on vascular calcification in 87 dialysis patients and observed a delay in VC progression [10]. Coincidentally, bisphosphonates, a type of mineralized inhibitor, have also been indicated to prevent hydroxyapatite formation by inhibiting calcium-phosphate crystal construction and soft tissue growth [11]. In patients undergoing dialysis, bisphosphonate administration for 12 months reduced coronary artery calcification progression but was associated with an increased risk of osteomalacia [12, 13]. Additionally, cinacalcet, a new drug recently incorporated into clinical practice for vascular calcification treatment, is used to treat secondary hyperparathyroidism by decreasing serum parathyroid hormone levels, thereby delaying the progression of vascular calcification [14]. The ADVANCE study, a multicentre trial conducted in dialysis patients, observed the effect of cinacalcet and reported a reduction in coronary artery calcification volume scores [15]. However, the above studies were limited by their small sample sizes and short follow-up times, making the conclusions restricted and reducing the generalizability. Moreover, there are few studies comparing the above three drugs with each other; thus, it cannot be determined which intervention is the most promising for treating vascular calcification.

Therefore, the aim of this study was to identify an appropriate intervention for vascular calcification among these different treatments. Conventional pairwise meta-analysis fails to analyse the associated merits of different treatments if they have not been examined in head-to-head trials. Network meta-analysis (NMA) allows comparisons to be inferred and then estimates the best approach. Thus, we conducted a network meta-analysis to compare the efficacy of sodium thiosulfate, bisphosphonates, and cinacalcet in terms of vascular calcification and provide a prospective strategy for the future.

## Methods

### Registration and protocol

The protocol of this network meta-analysis was prospectively registered on the International Prospective Register of Systematic Reviews (registration number: CRD42022379965). This network meta-analysis followed the PRISMA Extension Statement for Reporting of Systematic Reviews Incorporating Network Meta-analyses of Health Care Interventions (PRISMA-NMA) guidelines [16].

### Search strategy and literature source

Two investigators (LH and YZL) identified the studies through a systematic search of PubMed, Web of Science, the Cochrane Register of Controlled Trials databases, Embase and China National Knowledge Infrastructure (CNKI) databases from inception to November 2022, using the following search terms: vascular calcification, sodium thiosulfate, cinacalcet, bisphosphonates, randomized clinical trials (RCTs) and their medical subject heading (MeSH) terms with the Boolean search terms ‘OR’ and ‘AND’. The details of the search strategy were presented in the Supplementary Table 1.

### Inclusion and exclusion criteria

Two reviewers (L.H. and Y.Z.L.) independently screened the titles and abstracts then assessed the full texts of potentially relevant studies using EndNote 20. Disagreements were double-checked and resolved by a third reviewer (J.J.J.). Studies that met the following inclusion criteria were included in the subsequent analyses: (1) Studies that included patients (aged ≥ 18 years old) diagnosed with ESRD who were treated with regular haemodialysis for more than 3 months [17]. (2) Studies in which the patients in the treatment groups received a specific dose or duration of sodium thiosulfate, bisphosphonates or cinacalcet. Patients in the conventional therapy group used vitamin D supplements and phosphate binders. Other measures remained consistent between the two groups. (3) Studies in which at least one of the following results was mentioned: coronary artery calcification scores and aorta calcification scores (including abdominal aorta and iliac artery scores). In addition, each outcome indicator included pre- and posttreatment baseline values or differences. (4) Studies that were RCTs.

Studies were excluded if they met at least one of the following criteria: (1) studies including CKD patients without haemodialysis; (2) repeatedly published studies; (3) retrospective studies, case reports and case series, systematic evaluations and meta-analyses, reviews, and other literature related to non-RCT trials; (4) animal or cell studies; and (5) studies without full texts or with incomplete original data after contacting the authors.

### Data extraction and quality assessment

The following information was extracted from each study: author, publication year, baseline participant characteristics (country, age, sex etc.), interventions, trial duration and outcome measurements, such as the calcification score, serum calcium level, phosphorous level, and iPTH level. Based on the Cochrane handbook’s checklist of factors, a standardized data extraction form was created. If the original data only provided the median and quartile range, the web tool (https://www.math.hkbu.edu.hk/~tongt/papers/median2mean.html) was used to convert original data to mean and standard deviation. To overcome the differences of calcification scores between the conventional treatment group and the intervention group in baseline, we analyzed the data using the different value between the pre- and post-treatment calcification scores. ‘The Cochrane Collaboration’s tool for assessing risk of bias [18]’ was utilized to measure the risk of bias in the RCTs. RevMan 5.3 was used to represent the risk of bias. Two authors (L.H. and Y.Z.L.) independently graded the risk of bias in the studies and consulted the third author (J.J.J.) when discrepancies arose.

### Data synthesis and statistical analyses

Three key assumptions of a network meta-analysis include homogeneity, transitivity and consistency [19]. As interventions are by definition heterogeneous, pairwise meta-analyses on head-to-head comparisons based on the frequentist approach were executed. The standardized mean differences (SMD; Hedges’g) [20] and 95% intervals (CI) of vascular calcification score and serum indicators between the treatment and control groups were calculated. Furthermore, statistical heterogeneity was assessed using the statistic inconsistency index (I^2^). The fixed-effects model was used when I^2^ ≤ 50%, and the random-effects model was used when I^2^>50%.In pursuit of optimal accuracy of results, an I^2^ value greater than 50% is generally considered to indicate a substantial level of heterogeneity, which consequently initiates sensitivity analysis to identify the source [21]. In sensitivity analyses, we incorporated the risk of bias assessment by eliminating studies that were judged as having bias on the particular outcome. We used p values less than 0.05 to assess significance.

Transitivity is a key assumption of NMA and refers to the assumption that indirect comparison is an authentic estimate of the unobserved direct comparison [22]. In our study, we assessed connectivity of the network at different interventions visually and found no evidence of unconnectedness on either network. Another key assumption in network meta-analysis relates to consistency, however, there were no head-to-head RCTs included, all data were from indirect treatment comparisons, thus we were unable to assess statistical inconsistency [23].

We used STATA software (Version 17) to perform a multivariate network meta- analysis within a frequentist framework [24] according to current PRISMA NMA guidelines. The effective estimates were presented as a standardized mean difference (SMD) with a 95% confidence interval (CI). Results were considered as not statistically significant when 95% of CI contained null values. Evidence network diagrams were used to display the comparisons of different treatments. Connecting lines showed direct comparisons between the two interventions. The number of trials were indicated by the width of each line. The size of each node provided information about the intervention’s total sample size. Using the pairwise comparison, an interval plot was generated to present all possible pairwise comparisons between any two of the three treatments. The efficacy of each therapy was ranked using the surface under the cumulative ranking curve (SUCRA), expressed as a percentage with a range of 0 to 1. For efficacy assessment, interventions with highest SUCRA value corresponds to the most efficacious treatment. To assess the presence of bias due to small-scale studies, which could cause publication bias in NMA, funnel plots were created and visually inspected using the criterion of symmetry [25].

## Results

### Data extraction and quality assessment

**Fig. 1:**
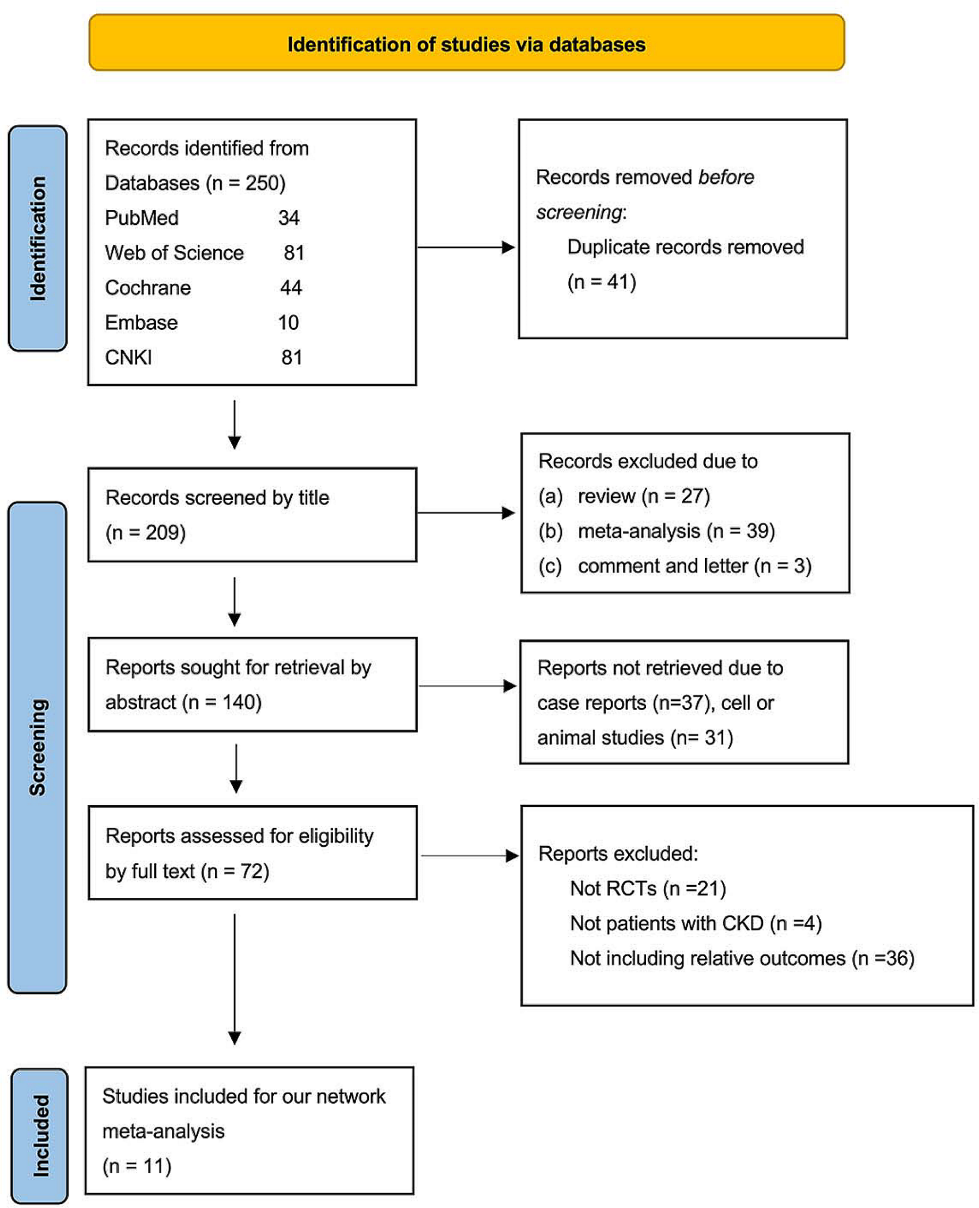
Flow chart showing the detailed procedures involved in study screening and the application of the exclusion criteria. Eleven studies were included in this network meta-analysis

**Fig. 2:**
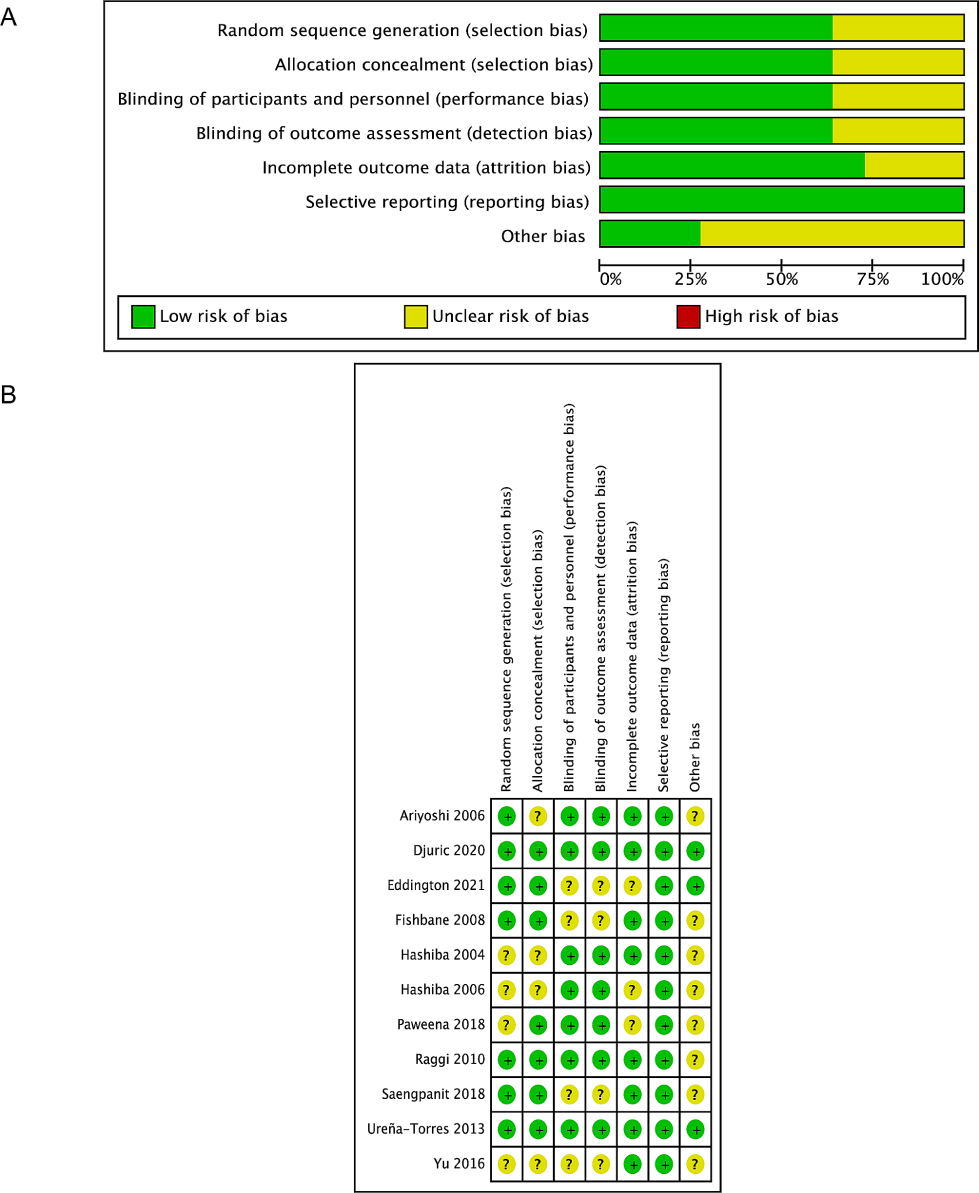
Quality assessment of the included literature. **(A)** Risk of bias graph; **(B)** risk of bias summary

In the present meta-analysis, a total of 250 studies were retrieved, of which 41 duplicated studies were eliminated. After reading the titles and abstracts, 137 studies that were reviews, meta-analyses, cell or animal studies, and case reports were excluded. Finally, after full-text review, eleven RCTs [12, 15, 26–34] met our criteria and were selected for our network meta-analysis. The flow diagram of literature identification and screening was shown in Fig. 1. The included studies had satisfactory qualities, as depicted in Fig. 2.

The clinical characteristics of the eleven included studies were provided in Table 1. In total, we enrolled 1083 participants, of which 543 patients were in treatment groups. In detail, these trials evaluated the efficacy of 3 different therapeutic agents, including sodium thiosulfate, bisphosphonates, and cinacalcet, for treating vascular calcification when compared to conventional therapeutic agents (vitamin D supplements and phosphate binders). The studies were conducted in Europe (*n* = 3) [26, 32, 33], Asia (*n* = 6) [12, 27–30, 34] and North America (*n* = 2) [15, 31]. For the study population, all eleven studies focused on patients treated with HD, and the average time of haemodialysis ranged from 7.02 to 104.4 months. Three studies reported the results of patients after treatment with sodium thiosulfate. Another three studies reported the use of 200–400 mg/day etidronate. The remaining five studies demonstrated that the utilization of cinacalcet started at 30 mg/d and was adjusted to achieve the targets. The time of treatment ranged from 3 to 12 months.

**Table 1:** Characteristics of studies included in this review.

Study ID	Year	Country	Study Design	Sample size		Age		Gender		Time of hemodialysis (months)		Intervention	Duration
				Treatment	Control	Treatment	Control	Treatment	Control	Treatment	Control		
Djuric, et al.	2020	Serbia	RCT	26	29	63.8 ± 13.2	64.1 ± 9.7	17 M,13 F	21 M,9 F	104.4 ± 80.5	103.7 ± 75.1	sodium thiosulfate 25 g/1.73 m^2^ iv three times a week	6 months
Saengpanit, et al.	2018	Thailand	RCT	24	25	50.4 ± 9.5	54.4 ± 10.7	12 M,12 F	16 M,10 F	72.3 ± 56.7	62 ± 55.8	sodium thiosulfate 12.5 g twice a week	6 months
Yu, et al.	2016	China	RCT	15	10	NA	NA	NA	NA	NA	NA	sodium thiosulfate 0.18 g/kg iv 3 times a week	3 months
Hashiba, et al.	2006	Japan	RCT	12	9	63.2 ± 3.33	67.7 ± 5.29	NA	NA	73.8 ± 22.7	63.6 ± 18.1	Etidronate 200 mg/day	12 months
Hashiba, et al.	2004	Japan	RCT	8	10	69.5 ± 3.2	62.1 ± 4.31	NA	NA	NA	NA	Etidronate 200 mg/day	6 months
Ariyoshi, et al.	2006	Japan	RCT	8	6	63.5 ± 8.3	66.3 ± 5.1	5 M,1 F	5 M,1 F	NA	NA	Etidronate 400 mg/day	6 months
Fishbane, et al.	2008	USA	RCT	87	86	57.7 ± 14.9	59.0 ± 12.4	52 M,35 F	45 M,41 F	46.3 ± 36.4	46.8 ± 44.1	Cinacalcet start with 30 mg/d, then adjusted to achieve targets	4 months
Ureña-Torres, et al.	2013	France	RCT	153	151	57.9 ± 13.6	57 ± 14.6	83 M,70 F	95 M,56 F	7.29 ± 2.54	7.02 ± 2.78	Cinacalcet start with 30 mg/d, then adjusted to achieve targets	12 months
Eddington, et al.	2021	UK	RCT	15	19	45 ± 16	54 ± 13	11 M,4 F	12 M,3 F	97.75 ± 89.97	103.75 ± 60.94	Cinacalcet start with 30 mg/d, then adjusted to achieve targets	12 months
Susantitaphong et al.	2019	Thailand	RCT	15	15	49.5 ± 11.9	49.4 ± 10.2	12 M,3 F	7 M,8 F	79.2 ± 43.2	84.0 ± 60.0	Cinacalcet start with 25 mg/d, then ajusted to achieve targets	3 months
Raggi, et al.	2011	USA	RCT	180	180	61.2 ± 12.6	61.8 ± 12.8	112 M,68 F	95 M,85 F	47.33 ± 17.72	47.73 ± 18.05	Cinacalcet start with 30 mg/d, then adjusted to achieve targets	12 months

### Intervention efficiency evaluation

#### Summary of evidence network

**Fig. 3:**
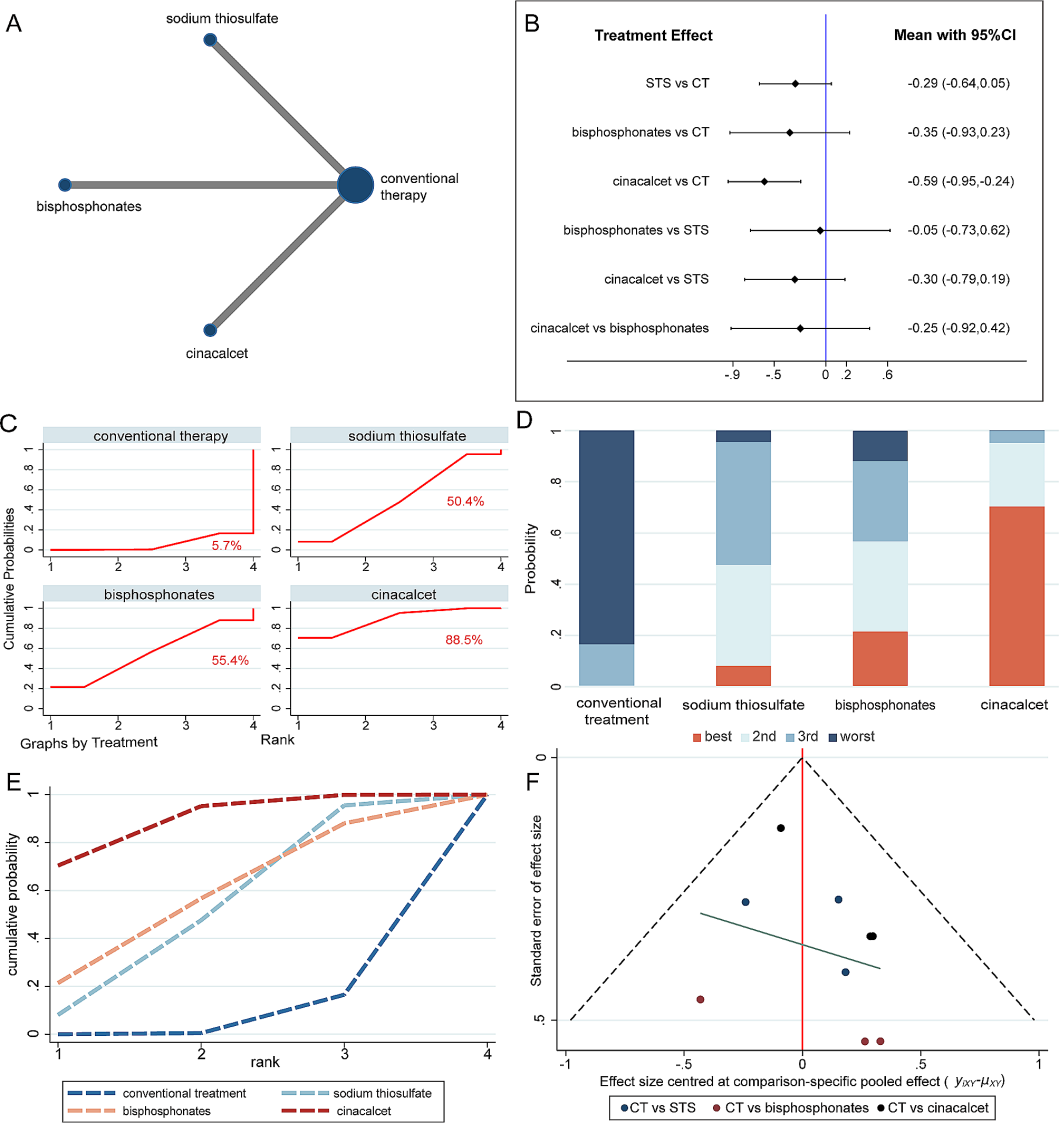
The network meta-analysis for calcification score. **(A)** Evidence network of all enrolled studies in relation to the calcification score of hemodialysis patients in this NMA; **(B)** Interval plots of network meta-analysis of calcification score; **(C)** The surface under the cumulative ranking curve (SUCRA) ranking chart for calcification score; **(D)** **(E)** ranking of calcification score of HD patients in this NMA. **(F)** funnel plot of calcification score. CT = conventional therapy; STS = sodium thiosulfate

**Fig. 4:**
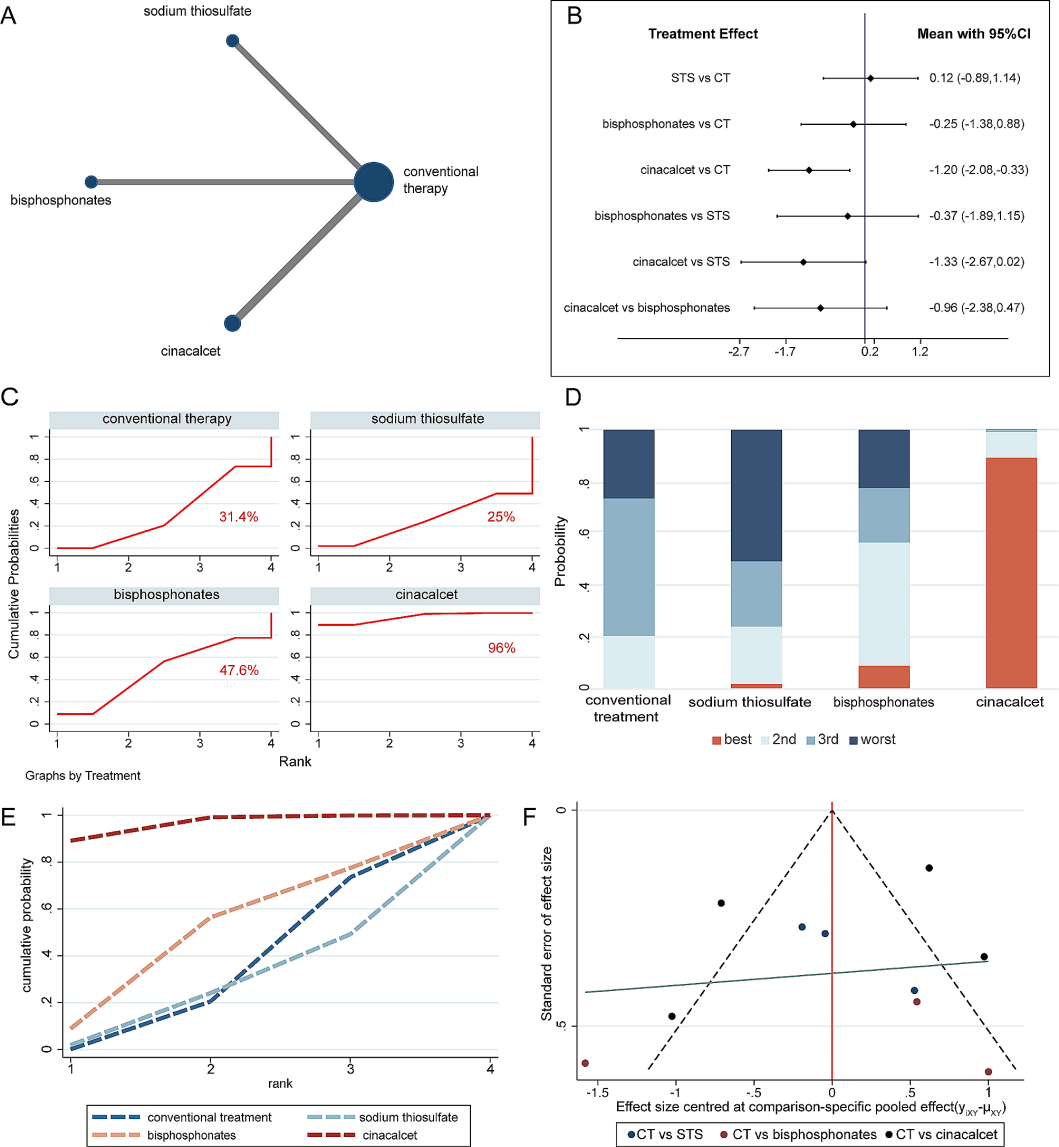
The network meta-analysis for serum calcium level. **(A)** Evidence network of all enrolled studies in relation to the serum calcium of hemodialysis patients in this NMA; **(B)** Interval plots of network meta-analysis of serum calcium; **(C)** The surface under the cumulative ranking curve (SUCRA) ranking chart for serum calcium; **(D)** **(E)** ranking of serum calcium of HD patients in this NMA. **(F)** funnel plot of serum calcium level. CT = conventional therapy; STS = sodium thiosulfate

**Fig. 5:**
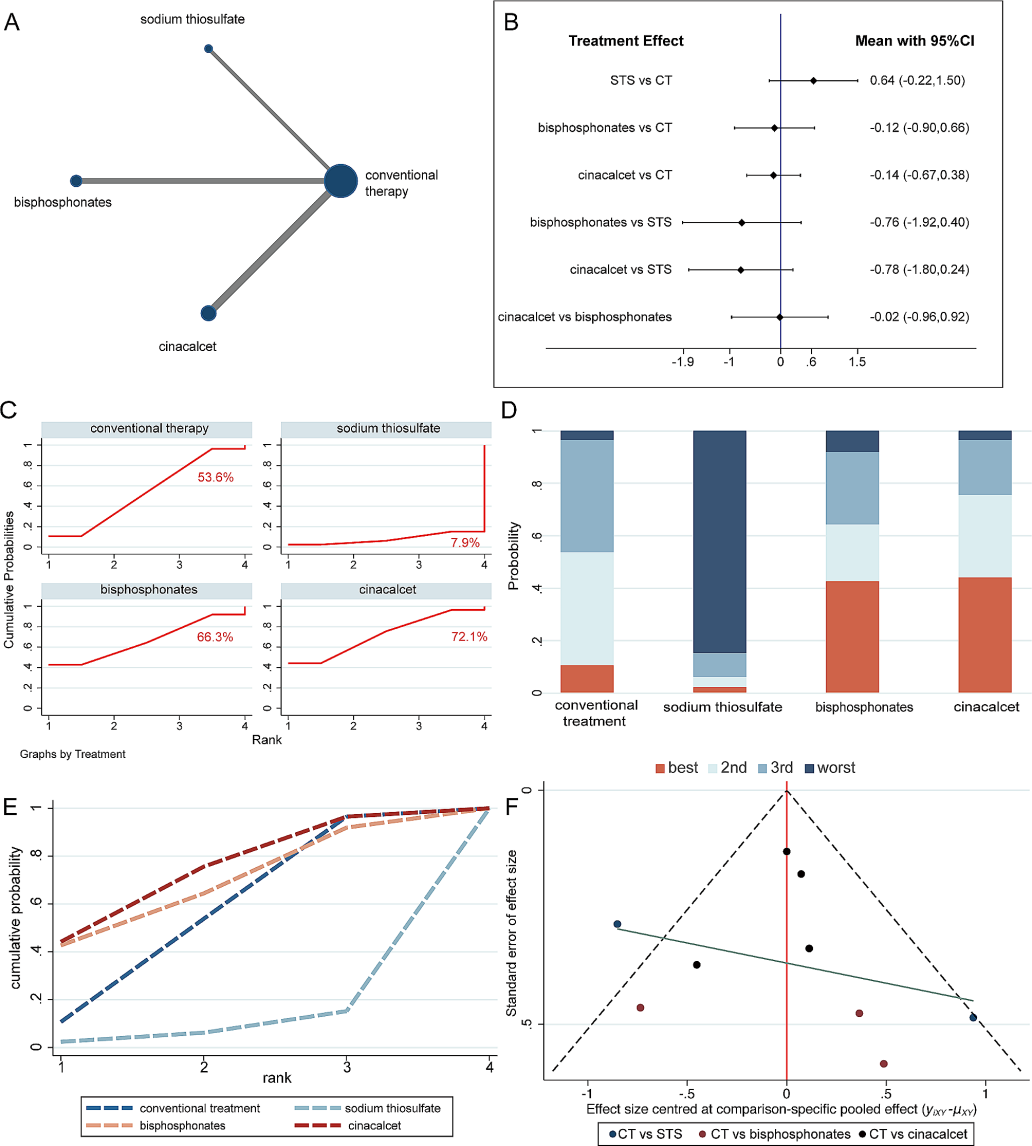
The network meta-analysis for serum phosphorus level. **(A)** Evidence network of all enrolled studies in relation to the serum phosphorus of hemodialysis patients in this NMA; **(B)** Interval plots of network meta-analysis of serum phosphorus; **(C)** The surface under the cumulative ranking curve (SUCRA) ranking chart for serum phosphorus; **(D)** **(E)** ranking of serum phosphorus of HD patients in this NMA. **(F)** funnel plot of serum phosphorus level. CT = conventional therapy; STS = sodium thiosulfate

**Fig. 6:**
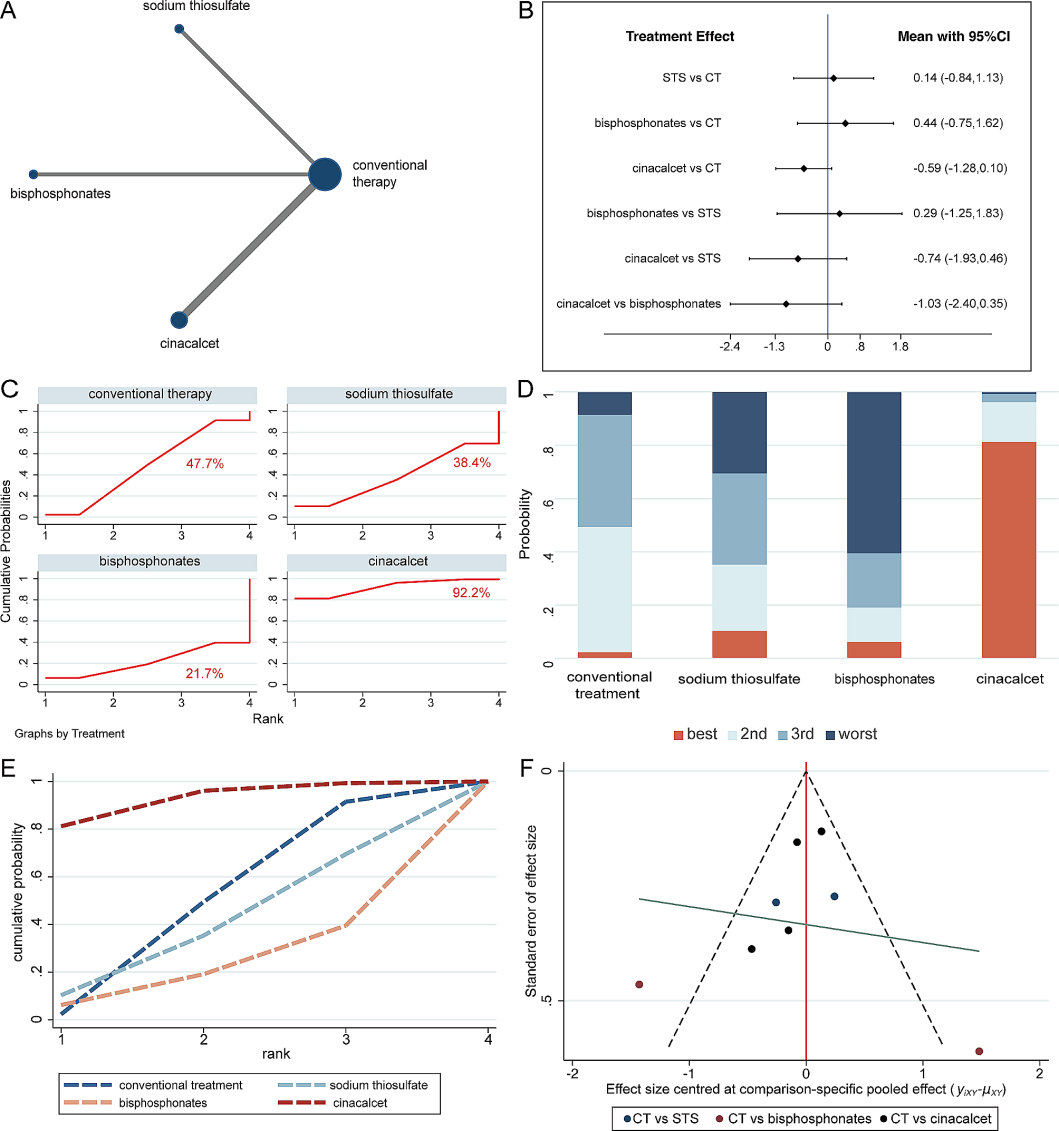
The network meta-analysis for serum intact Parathyroid Hormone, (iPTH) level. **(A)** Evidence network of all enrolled studies in relation to the serum iPTH of hemodialysis (HD) patients in this NMA; **(B)** Interval plots of network meta-analysis of serum iPTH; **(C)** The surface under the cumulative ranking curve (SUCRA) ranking chart for serum iPTH; **(D)** **(E)** ranking of serum phosphorus of HD patients in this NMA. **(F)** funnel plot of serum iPTH level. CT = conventional therapy; STS = sodium thiosulfate

The evidence networks displayed in Figs. 3A, 4A and 5A, and 6A indicated the correlation of sodium thiosulfate, bisphosphonates and cinacalcet with conventional therapy across the 3 arms. Since the original studies evaluated the effectiveness of these drugs separately when compared to conventional therapy, there was no pairwise comparison of the three drugs. In other words, the current network meta-analysis had no triangular loop. Thus, there was no source of inconsistency in our analysis.

#### Effect of different interventions on the calcification score

Six studies included 509 patients undergoing haemodialysis and provided data on calcification scores. Pairwise analyses demonstrated that compared with conventional therapy, there was a significant attenuation of vascular calcification after treatment with cinacalcet (SMD: −0.60, 95%CI: [− 0.83, − 0.37], *P* = 0.000, I^2^ = 0.00%, Supplementary Fig. 1C). Whereas sodium thiosulfate (SMD − 0.29, 95%CI: [− 0.64, 0.05], *P* = 0.091, I^2^ = 0.00%, Supplementary Fig. 1A) and bisphosphonates (SMD: −0.24, 95%CI: [− 0.81, 0.34], *P* = 0.369, I^2^ = 0.00%, Supplementary Fig. 1B) did not display any mitigation of vascular calcification when comparing with conventional therapy.

In agreement with the pairwise meta-analysis, the results of the interval plot demonstrated that cinacalcet (SMD − 0.59; 95% CI [− 0.95, − 0.24]) remarkably mitigated vascular calcification compared with conventional therapy in haemodialysis patients (Fig. 3B). Furthermore, cinacalcet showed a tendency to be more effective than sodium thiosulfate (SMD − 0.29; 95% CI [− 0.64, 0.05]) and bisphosphonates (SMD − 0.35; 95% CI [− 0.93, 0.23]) in alleviating vascular calcification (Fig. 3B). For ranking the efficacy assessment, cinacalcet had the highest SUCRA value (88.5%) for alleviating vascular calcification (Fig. 3C-E). The above results suggested that cinacalcet possessed more therapeutic prospects for vascular calcification. The funnel plot did not suggest that there was any publication bias or other small study effects (Fig. 3F).

#### Effect of different interventions on serum calcium levels

Additionally, analyses were performed to explore the different drug therapeutic effects on serological markers associated with vascular calcification. Ten trials with a total of 723 patients undergoing haemodialysis reported the level of serum calcium. The forest plots displayed the efficacy of different treatment compared with the conventional therapy. There was a significant serum calcium reduction in cinacalcet group (SMD: −1.20, 95%CI: [− 2.09, − 0.31], *P* = 0.008, I^2^ = 92.4%, Supplementary Fig. 2C) but neither sodium thiosulfate (SMD: 0.04, 95%CI: [− 0.31, 0.39], *P* = 0.823, I^2^ = 5.7%, Supplementary Fig. 2A) nor bisphosphonates (SMD: −0.25, 95%CI: [− 1.72, 1.21], *P* = 0.734, I^2^ = 82.4%, Supplementary Fig. 2B) demonstrated such an effect.

Based on the pairwise analysis, we conducted the network meta-analysis. The outcome revealed that cinacalcet (SMD − 1.20; 95% CI [–2.08, − 0.33]) was superior in reducing serum calcium levels compared with conventional therapy (Fig. 4B), while the other two therapies were not statistically significant. Moreover, cinacalcet showed a tendency to be more effective than sodium thiosulfate (SMD − 1.33; 95% CI [–2.67, 0.02]) and bisphosphonates (SMD − 0.96; 95% CI [–2.38, 0.47]) in reducing serum calcium levels. Considering the efficiency of lowering serum calcium levels, remarkably, cinacalcet had the highest SUCRA value (96.0%), followed by bisphosphonates (47.6%) and conventional treatment (31.4%) (Fig. 4C-E). Therefore, cinacalcet was rated as the best treatment for reaching the standard level of serum calcium. The funnel plot indicated that some points fell outside the 95% CI, which may be related to the heterogeneity in the study (Fig. 4F).

#### Effect of different interventions on serum phosphorus levels

Nine studies published relevant data on serum phosphorus levels in 668 haemodialysis patients. As shown by pairwise meta-analyses versus conventional treatment (Supplementary Fig. 3A-C), sodium thiosulfate (SMD: 0.78, 95%CI: [− 0.97, 2.53], *P* = 0.384, I^2^ = 90.1%) nor bisphosphonates (SMD: −0.15, 95%CI: [− 0.72, 0.41], *P* = 0.596, I^2^ = 47%) and cinacalcet group (SMD: −0.10, 95%CI: [− 0.29, 0.09], *P* = 0.319, I^2^ = 0.0%) all failed to lower blood phosphorus.

Next, network meta-analysis was performed. The result of network meta-analysis indicated that the efficacy of sodium thiosulfate (SMD 0.64; 95% CI [–0.22, 1.50]), bisphosphonates (SMD − 0.12; 95% CI [–0.9, 0.66]), and cinacalcet (SMD − 0.14; 95% CI [–0.67, 0.38]) in decreasing serum phosphorus levels was similar to that of conventional therapy (Fig. 5B). There was no significant difference in the comparison of the interventions with each other. The corresponding SUCRA values are presented in Fig. 5C, and the comparative ranking of SUCRA values is shown in Fig. 5D-E. The top three SUCRA values were 72.1%, 66.3% and 53.6% for cinacalcet, bisphosphonates and conventional therapy, respectively. As for evaluating publication bias, the funnel plot appeared symmetrical (Fig. 5F).

#### Effect of different interventions on serum iPTH levels

Secondary hyperparathyroidism (SHPT) is considered as a vital factor in the development of vascular calcification. Therefore, we further evaluated the effect of these three interventions on serum iPTH levels. The incidence of serum iPTH level was showed from eight trials with 654 patients on haemodialysis. In pairwise comparisons, cinacalcet (SMD: −0.47, 95%CI: [− 0.65, − 0.29], *P* = 0.00, I^2^ = 0.00%, Supplementary Fig. 4C) exert reducing serum iPTH levels than conventional therapy. Nevertheless, sodium thiosulfate (SMD: 0.15 95%CI: [− 0.23, 0.54], *P* = 0.434, I^2^ = 37.6%, Supplementary Fig. 4A) and bisphosphonates (SMD: 0.57, 95%CI: [− 2.28, 3.42], *P* = 0.697, I^2^ = 93%, Supplementary Fig. 4B) didn’t demonstrated advantage over conventional treatment.

Although there was no significant difference observed between sodium thiosulfate, bisphosphonates and cinacalcet and conventional therapy, cinacalcet (SMD − 0.59; 95% CI [–1.28, 0.10]) was the only intervention that tended to decrease the iPTH level (Fig. 6B). Moreover, cinacalcet still showed a tendency to lower iPTH level when compared with sodium thiosulfate (SMD − 0.74; 95% CI [–1.93, 0.46]) and bisphosphonates (SMD − 1.03; 95% CI [–2.40, 0.35]). Figure 6C-E illustrated the rank probability of the efficacy of different medications on decreasing serum iPTH levels, suggesting that cinacalcet was most likely to be ranked as the first therapeutic option (92.2%). The funnel plots of the network meta-analysis for iPTH were not suggestive of publication bias (Fig. 6F).

#### Sensitivity analyses

Furthermore,sensitivityanalyseswere performed to ensure robustness of results (Supplementary Fig. 5). After removing studies one by one, the ranking outcomes of network meta-analyses were similar to the original studies among serum calcium level and serum iPTH level. When excluding Raggi in the sensitivity analysis of vascular calcification score, we found that cinacalcet (SUCRA:72.3%) remained the most favorable treatment for alleviating vascular calcification (Supplementary Fig. 6).

## Discussion

To the best of our knowledge, this network meta-analysis is the first to synthesize evidence for evaluating the impact of sodium thiosulfate, bisphosphonates and cinacalcet on vascular calcification. Notably, the results of network meta-analysis indicated that compared with conventional therapy, cinacalcet could significantly reduce vascular calcification in haemodialysis patients. In addition, the most striking result indicated that cinacalcet was superior to sodium thiosulfate and bisphosphonates in delaying the process of vascular calcification. Mechanistically, our findings illustrated that the maintenance of stable calcium and iPTH levels might be the main reason for the effect of cinacalcet on reducing vascular calcification. Overall, our outcomes demonstrated that cinacalcet is a promising choice of treatment for vascular calcification in patients with haemodialysis.

Cinacalcet is a calcimimetic compound that acts on the calcium sensing receptor (CaSR) and subsequently inhibits parathyroid hormone secretion [35, 36]. Currently, cinacalcet is widely used to treat moderate and severe secondary hyperparathyroidism in dialysis patients. A prospective cohort study conducted in Japan with 47 patients demonstrated a reduction in abdominal aortic calcification over a 12-month period of cinacalcet treatment in the real world [37]. Moreover, in in vitro experiments, Wu et al. illustrated significant aortic calcification attenuation in uraemic rats that were orally administered cinacalcet for 12 weeks [38]. Consistently, our systematic review of large and comprehensive RCTs also revealed that cinacalcet delayed the process of vascular calcification relative to conventional therapy.

In addition, our analysis suggested that cinacalcet was better than sodium thiosulfate and bisphosphonates in retarding vascular calcification, which was confirmed by its high SUCRA values. Regarding sodium thiosulfate, Mathews et al. treated 22 HD patients with intravenous sodium thiosulfate for 5 months and reported no significant differences in the mean annualized changeable rates of the calcium volume in aorta, coronary, or vertebral bone density [39]. Although it has been proposed that sodium thiosulfate can chelate calcium to form highly soluble calcium thiosulfate salt [40], this process seems to be unreasonable because sodium thiosulfate cannot lower circulating calcium levels [14]. To date, bisphosphonates have also been used to alleviate vascular calcification. Nevertheless, in the CKD stage 3–4 population, 18 months of bisphosphonate usage failed to lead to a difference in the progression of aortic vascular calcification [41]. Furthermore, the safety of bisphosphonates regarding long-term use is unclear, and researchers are still concerned about the possible risk of exacerbated adynamic bone disease and osteomalacia [42]. However, the bisphosphonate included in the present study is etidronate only, and these results may not be generalizable to other bisphosphonates. In summary, neither drug led to a reduction in vascular calcification. Consistent with the above studies, our findings demonstrated that neither sodium thiosulfate nor bisphosphonates significantly mitigated vascular calcification, while cinacalcet exhibited such an effect. Therefore, cinacalcet might be the best recommendation for dialysis patients with potentially progressive vascular calcification.

Cinacalcet mimics the action of calcium by allosterically activating CaSR on the chief cell of the parathyroid gland to directly suppress PTH secretion and indirectly reduce serum calcium levels [43]. Joki et al. evaluated the role of calcimimetics in uraemic mice and emphasized that the activation of CaSR might contribute to slowing the progression of vascular calcification [44]. In addition, Kawata et al. found that cinacalcet markedly lessened calcification-related changes by reducing serum parathyroid hormone and calcium levels in rats with a remnant kidney model of uraemia [45]. Similarly, clinical treatment with cinacalcet lowered serum calcium and phosphate levels, thereby slowing the progression of cardiovascular calcification [46]. In summary, cinacalcet is now successfully used in conjunction with phosphate binders and active vitamin D in the treatment of SHPT and vascular calcification in dialysis patients. However, the side effects associated with cinacalcet should also be brought up as high importance. The results of a meta-analysis suggested that cinacalcet significantly increased the risk of hypocalcaemia, nausea, and vomiting [47], and Xu et al. found out that gastrointestinal events were noted at greater doses [48]. These side effects may be correlated with the pharmacological action and therefore need to be closely monitored during the use of cinacalcet.

Recent studies have indicated that the cardiovascular benefits of cinacalcet are also worth noting. Cunningham et al. analysed the clinical data of four RCTs and demonstrated that cinacalcet decreased the risk of cardiovascular hospitalization [49]. In addition, the post hoc analysis of the EVOLVE trial, which was conducted in 3883 dialysis patients with a 2-year follow-up, showed a lower incidence of calcific uraemic arteriolopathy [50], a tendency towards a reduced fracture rate [51], and a decreased risk of cardiovascular events in patients over the age of 65 years [52]. Overall, the cardiovascular benefit demonstrated by cinacalcet might be associated with a reduction in vascular calcification.

The follow-up time in the studies included in the present meta-analysis is relatively short (ranging from 3 to 12 months). However, the hemodialysis patients we included already had vascular calcification at the baseline of the studies. In our analysis, our focus is on whether vascular calcification has progressed before and after these drugs treatment. And the follow-up time in all studies included in the present meta-analysis is similar. Hence, its influence on the results is limited. Another question worth considering is that the drug dosages and durations were not entirely consistent among the studies we included. However, the pairwise meta-analysis revealed no significant heterogeneity in the combination of the same drugs. And the sensitivity analyses showed that the results of network meta-analyses were stable. Therefore, we thought our network meta-analyses had certain clinical value to roughly compare the efficacy of these three drugs. Certainly, different doses of intervention and durations of treatment made some limitations for the results, and further direct RCT research is needed in the future to prove our results of NMA.

There are several potential limitations in this network meta-analysis. First, due to the lack of direct RCTs of treatment comparisons, consistency was unable to assess. However, after excluding studies may potentially lead to heterogeneity, the results of NMA did not materially change for the primary outcome, which hinted at the stability of our findings. For sure, further direct comparative trials for sodium thiosulfate, bisphosphonates, and cinacalcet are needed to verify our conclusion. Second, in the hemodialysis population, the disturbances of calcium and phosphorus metabolism induces systemic vascular calcification, and the progression of vascular calcification may vary among different sites. This variation may affect the evaluation of drug efficacy, leading to a certain bias in our analysis and further homogeneity research is needed to confirm our results. Third, our study was conducted with haemodialysis patients but did not assess the underlying ability of these drugs in the chronic kidney disease population with renal transplantation or peritoneal dialysis. Certainly, further large-scale and head-to-head studies should be conducted to confirm the generalizability of the present results.

## Conclusion

This network meta-analysis indicated that cinacalcet appear to be more effective than sodium thiosulfate and bisphosphonates in mitigating vascular calcification through decreasing serum calcium and iPTH. And cinacalcet might be a reasonable option for hemodialysis patients with VC in clinical practice. Certainly, further large-scale or head-to-head RCTs are required to verify these conclusions.

### Electronic supplementary material

Below is the link to the electronic supplementary material.

Supplementary Material 1

## Data Availability

The original data presented in the study are publicly available as cited in the reference section, further requirements can be directed to the corresponding author.
