# Supplementary material for: Comparative efficacy of sodium thiosulfate, bisphosphonates, and cinacalcet for the treatment of vascular calcification in patients with haemodialysis: a systematic review and network meta-analysis
Source: BMC Nephrol. 2024 Jan 22;25:26. doi: 10.1186/s12882-024-03460-x (PMC10804723; doi:10.1186/s12882-024-03460-x)
Supplement: Supplementary file 1 — Supplementary Material 1 [file 12882_2024_3460_MOESM1_ESM.docx]

Supplement Material

# Supplementary Tables

### Supplement Table 1. Search strategy

| Data base | Search Term |
| --- | --- |
| Pubmed | #1: ((("sodium, dietary"[MeSH Terms] OR ("sodium"[All Fields] AND "dietary"[All Fields]) OR "dietary sodium"[All Fields] OR "sodium"[All Fields] OR "sodium"[MeSH Terms] OR "sodiums"[All Fields]) AND "thiosulfates"[MeSH Terms]) OR (("thiosulfuric"[All Fields] OR "thiosulphuric"[All Fields]) AND "acid disodium salt"[Title/Abstract]) OR "sodium thiosulfate pentahydrate"[Title/Abstract] OR (("sodium thiosulfate"[Supplementary Concept] OR "sodium thiosulfate"[All Fields]) AND "USAN"[Title/Abstract]) OR ("pentahydrate"[All Fields] AND "sodium thiosulfate"[Title/Abstract]) OR (("sodium thiosulfate"[Supplementary Concept] OR "sodium thiosulfate"[All Fields]) AND "anhydrous"[Title/Abstract]))  #2:("Diphosphonates"[MeSH Terms] OR ("bisphosphonated"[All Fields] OR "bisphosphonic"[All Fields] OR "Diphosphonates"[MeSH Terms] OR "Diphosphonates"[All Fields] OR "bisphosphonate"[All Fields] OR "bisphosphonates"[All Fields]) OR ("bisphosphonated"[All Fields] OR "bisphosphonic"[All Fields] OR "Diphosphonates"[MeSH Terms] OR "Diphosphonates"[All Fields] OR "bisphosphonate"[All Fields] OR "bisphosphonates"[All Fields]))  #3:("Cinacalcet"[MeSH Terms] OR ("Cinacalcet"[MeSH Terms] OR "Cinacalcet"[All Fields] OR ("Cinacalcet"[All Fields] AND "hydrochloride"[All Fields]) OR "cinacalcet hydrochloride"[All Fields]) OR (((("alpha methyl n"[All Fields] AND (("3"[All Fields] AND (("3"[All Fields] AND ("trifluoromethyl"[All Fields] OR "trifluoromethylated"[All Fields] OR "trifluoromethylating"[All Fields] OR "trifluoromethylation"[All Fields] OR "trifluoromethylations"[All Fields] OR "trifluoromethylative"[All Fields])) AND ("phenyl"[All Fields] OR "phenylated"[All Fields] OR "phenylation"[All Fields] OR "phenylic"[All Fields] OR "phenyls"[All Fields]))) AND ("propyl"[All Fields] OR "propylated"[All Fields] OR "propylation"[All Fields] OR "propylic"[All Fields]))) AND "1 naphthalenemethanamine"[All Fields]) AND "alphaR"[All Fields]) AND ("hydrochlorid"[All Fields] OR "hydrochloride"[All Fields] OR "hydrochlorides"[All Fields])) OR ("Cinacalcet"[MeSH Terms] OR "Cinacalcet"[All Fields] OR "sensipar"[All Fields]) OR ("Cinacalcet"[MeSH Terms] OR "Cinacalcet"[All Fields] OR "amg 073"[All Fields]) OR ("Cinacalcet"[MeSH Terms] OR "Cinacalcet"[All Fields] OR "krn 1493"[All Fields]) OR ("Cinacalcet"[MeSH Terms] OR "Cinacalcet"[All Fields] OR "amg073"[All Fields]))  #4: ("Vascular Calcification"[MeSH Terms] OR "calcification vascular"[Title/Abstract] OR "vascular calcifications"[Title/Abstract] OR "vascular calcinosis"[Title/Abstract] OR (("Calcinosis"[MeSH Terms] OR "Calcinosis"[All Fields] OR "Calcinoses"[All Fields]) AND "Vascular"[Title/Abstract]) OR (("Calcinosis"[MeSH Terms] OR "Calcinosis"[All Fields] OR "Calcinoses"[All Fields]) AND "Vascular"[Title/Abstract]) OR (("blood vessels"[MeSH Terms] OR ("blood"[All Fields] AND "vessels"[All Fields]) OR "blood vessels"[All Fields] OR "Vascular"[All Fields] OR "neovascularization, pathologic"[MeSH Terms] OR ("neovascularization"[All Fields] AND "pathologic"[All Fields]) OR "pathologic neovascularization"[All Fields] OR "vascularisation"[All Fields] OR "vascularization"[All Fields] OR "vascularisations"[All Fields] OR "vascularise"[All Fields] OR "vascularised"[All Fields] OR "vascularities"[All Fields] OR "vascularitis"[All Fields] OR "vascularity"[All Fields] OR "vascularizations"[All Fields] OR "vascularize"[All Fields] OR "vascularized"[All Fields] OR "vascularizes"[All Fields] OR "vascularizing"[All Fields] OR "vasculars"[All Fields]) AND "Calcinoses"[Title/Abstract]))  #5: ("Randomized Controlled Trials as Topic"[MeSH Terms] OR "randomized controlled trial"[Publication Type])  #6: #1AND #4 AND #5  #7: #2AND #4 AND #5  #8: #3AND #4 AND #5 |
| Embase | #1: ('sodium thiosulfate':ab,ti OR 'thiosulfuric acid, disodium salt':ab,ti OR 'sodium thiosulfate pentahydrate':ab,ti OR 'sodium thiosulfate':ab,ti OR 'pentahydrate of sodium thiosulfate':ab,ti OR 'sodium thiosulfate anhydrous':ab,ti)  #2: (diphosphonates:ab,ti OR bisphosphonates:ab,ti OR bisphosphonate:ab,ti)  #3: (cinacalcet:ab,ti OR 'cinacalcet hydrochloride':ab,ti OR 'alpha-methyl-n-3-3-trifluoromethyl phenyl propyl-1-naphthalenemethanamine, alphar-hydrochloride':ab,ti OR sensipar:ab,ti OR 'amg 073':ab,ti OR 'krn 1493':ab,ti OR amg073:ab,ti)  #4: ('vascular calcification':ab,ti OR 'calcification, vascular':ab,ti OR 'vascular calcifications':ab,ti OR 'vascular calcinosis':ab,ti OR 'calcinoses, vascular':ab,ti OR 'calcinosis, vascular':ab,ti OR 'vascular calcinoses':ab,ti)  #5: 'randomized controlled trials':ab,ti  #6: #1AND #4 AND #5  #7: #2AND #4 AND #5  #8: #3AND #4 AND #5 |
| Web of science | #1: TS=(sodium thiosulfate OR thiosulfuric acid, disodium salt OR sodium thiosulfate pentahydrate OR sodium thiosulfate OR pentahydrate of sodium thiosulfate OR sodium thiosulfate anhydrous)  #2: TS=( Diphosphonates OR Bisphosphonates OR Bisphosphonate)  #3: TS=(Cinacalcet OR Cinacalcet Hydrochloride OR Alpha-methyl-N-3-3-trifluoromethyl phenyl propyl-1-naphthalenemethanamine, alphaR-hydrochloride OR Sensipar OR AMG 073 OR KRN 1493 OR AMG073)  #4: TS=(Vascular Calcification OR Calcification, Vascular OR Vascular Calcifications OR Vascular Calcinosis OR Calcinoses, Vascular OR Calcinosis, Vascular OR Vascular Calcinoses)  #5: TS=(Randomized Controlled Trials)  #6: #1AND #4 AND #5  #7: #2AND #4 AND #5  #8: #3AND #4 AND #5 |
| The Cochrane | #1: Thiosulfate, Sodium Gold OR Aurothiosulfate, Sodium OR Bis monothiosulfato 2O,S aurate3 Trisodium OR Sodium Thiosulfate, Gold OR Sodium Gold Thiosulfate OR Thiosulfate, Gold Sodium OR Sodium Aurothiosulfate OR Gold Thiosulfate, Sodium OR Thiosulfatoaurate OR Sanochrysine OR Sanochrysin OR Sanocrisin  #2: Bisphosphonate OR Bisphosphonates  #3: Cinacalcet OR Alpha methyl N 3 3 trifluoromethylphenylpropyl 1 naphthalenemethanamine, alphaR hydrochloride OR Cinacalcet Hydrochloride OR Sensipar OR KRN 1493 OR AMG073 OR AMG 073  #4: Vascular Calcinosis OR Calcinosis, Vascular OR Calcinoses, Vascular OR Calcification, Vascular OR Vascular Calcifications OR Calcifications, Vascular OR Vascular Calcinoses  #5: Randomized Controlled Trial  #6: #1AND #4 AND #5  #7: #2AND #4 AND #5  #8: #3AND #4 AND #5 |

# Supplementary Figures


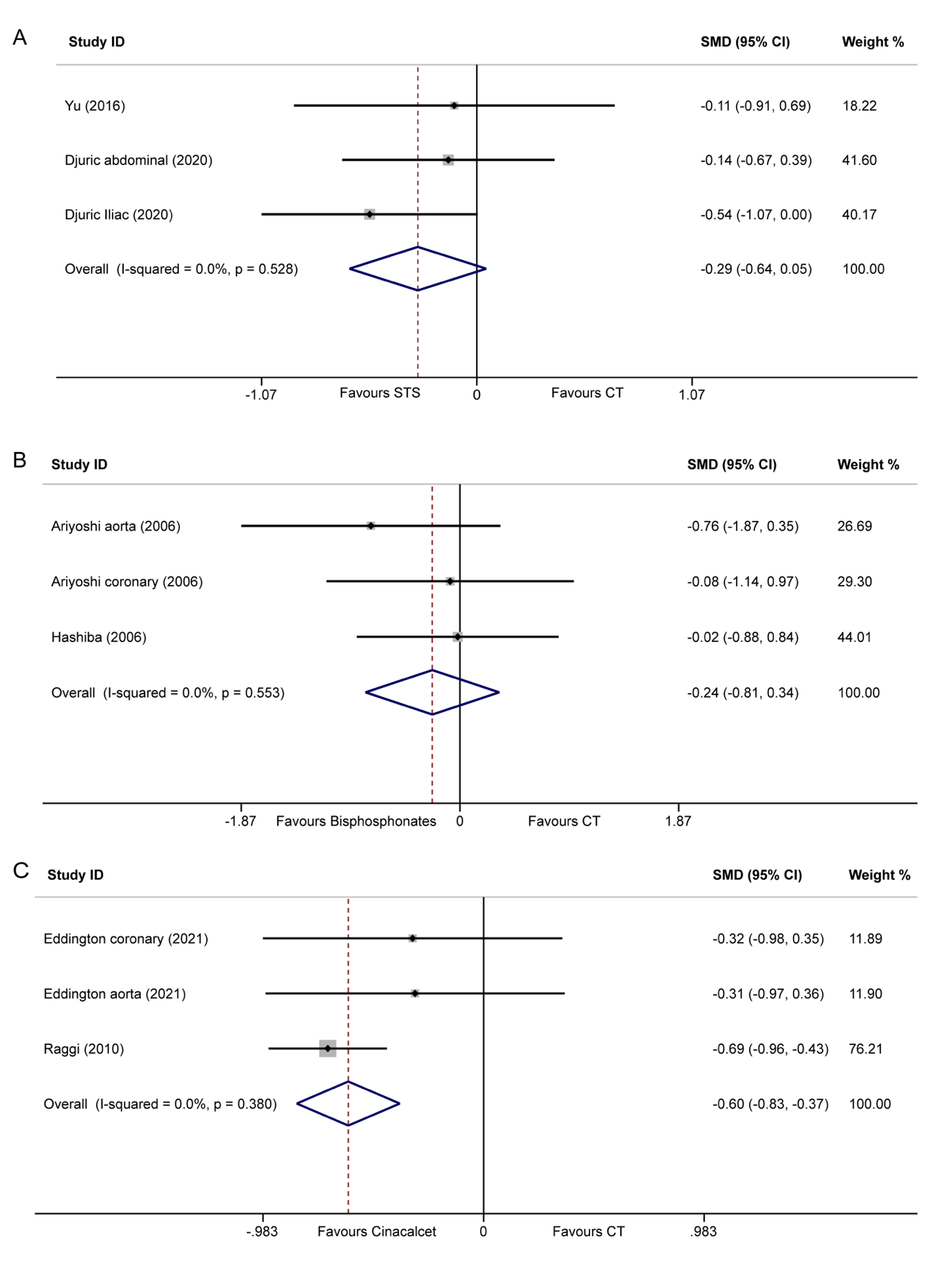


**Supplement Figure 1.** **Meta-analysis of** **calcification score.** (A) Forest plots of sodium thiosulfate; (B) Forest plots of bisphosphonates; (C) Forest plots of cinacalcet. CT= conventional therapy; STS= sodium thiosulfate.

#
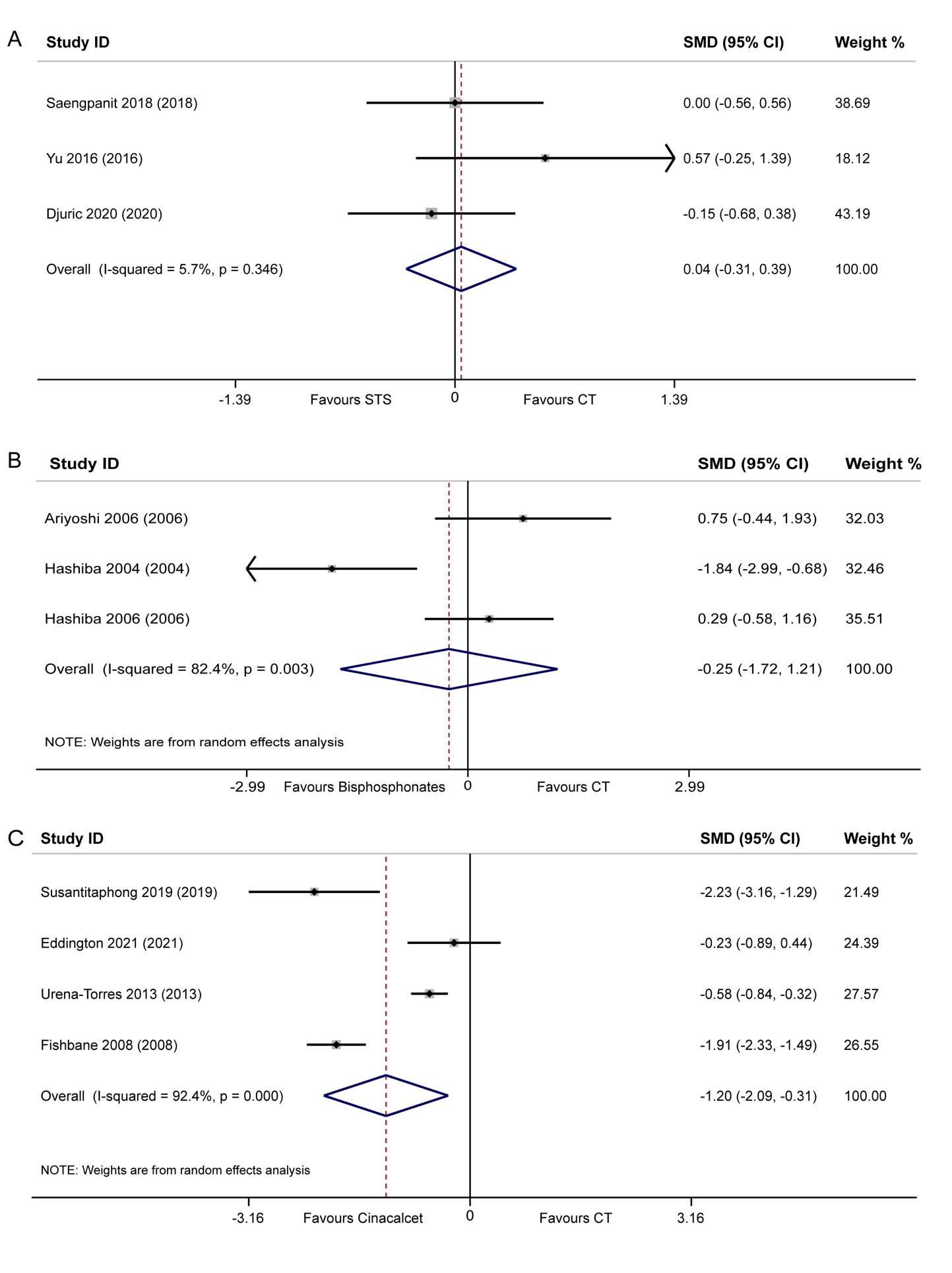
Supplement Figure 2. Meta-analysis of serum calcium level. (A) Forest plots of sodium thiosulfate; (B) Forest plots of bisphosphonates; (C) Forest plots of cinacalcet. CT= conventional therapy; STS= sodium thiosulfate.


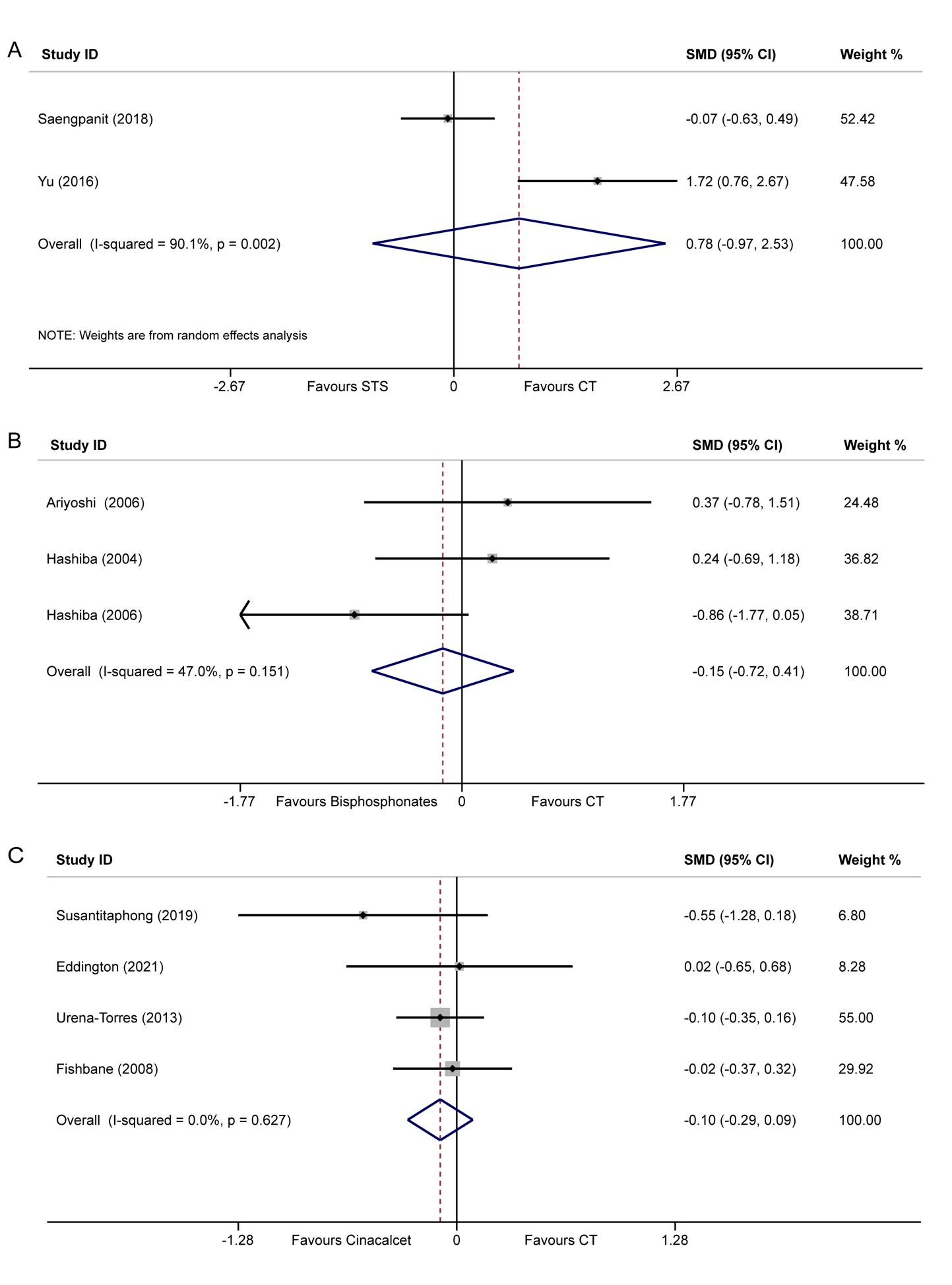


**Supplement Figure 3. Meta-analysis of serum phosphorus level.** (A) Forest plots of sodium thiosulfate; (B) Forest plots of bisphosphonates; (C) Forest plots of cinacalcet. CT= conventional therapy; STS= sodium thiosulfate.


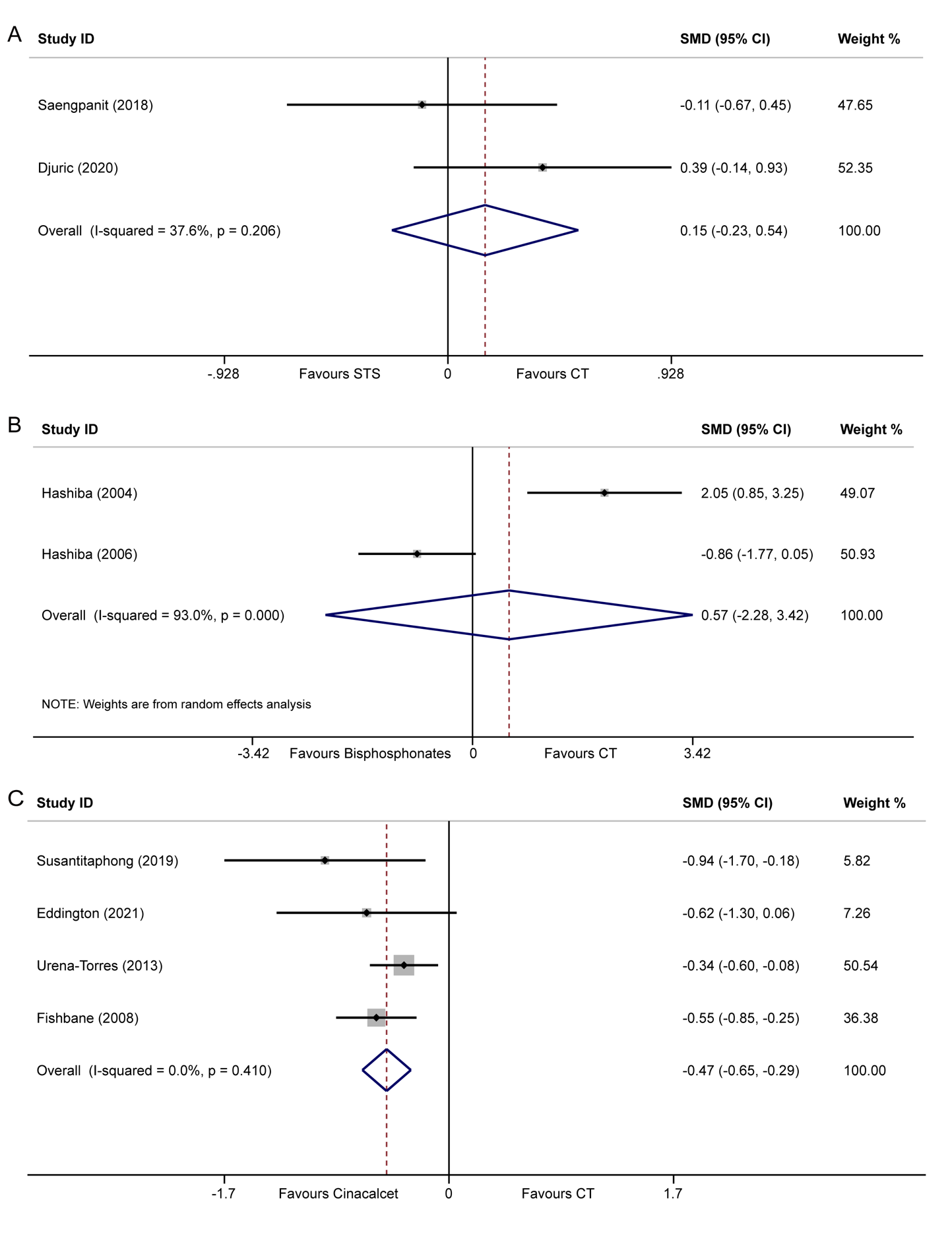


**Supplement Figure 4. Meta-analysis of** **serum iPTH level.** (A) Forest plots of sodium thiosulfate; (B) Forest plots of bisphosphonates; (C) Forest plots of cinacalcet. CT= conventional therapy; STS= sodium thiosulfate.


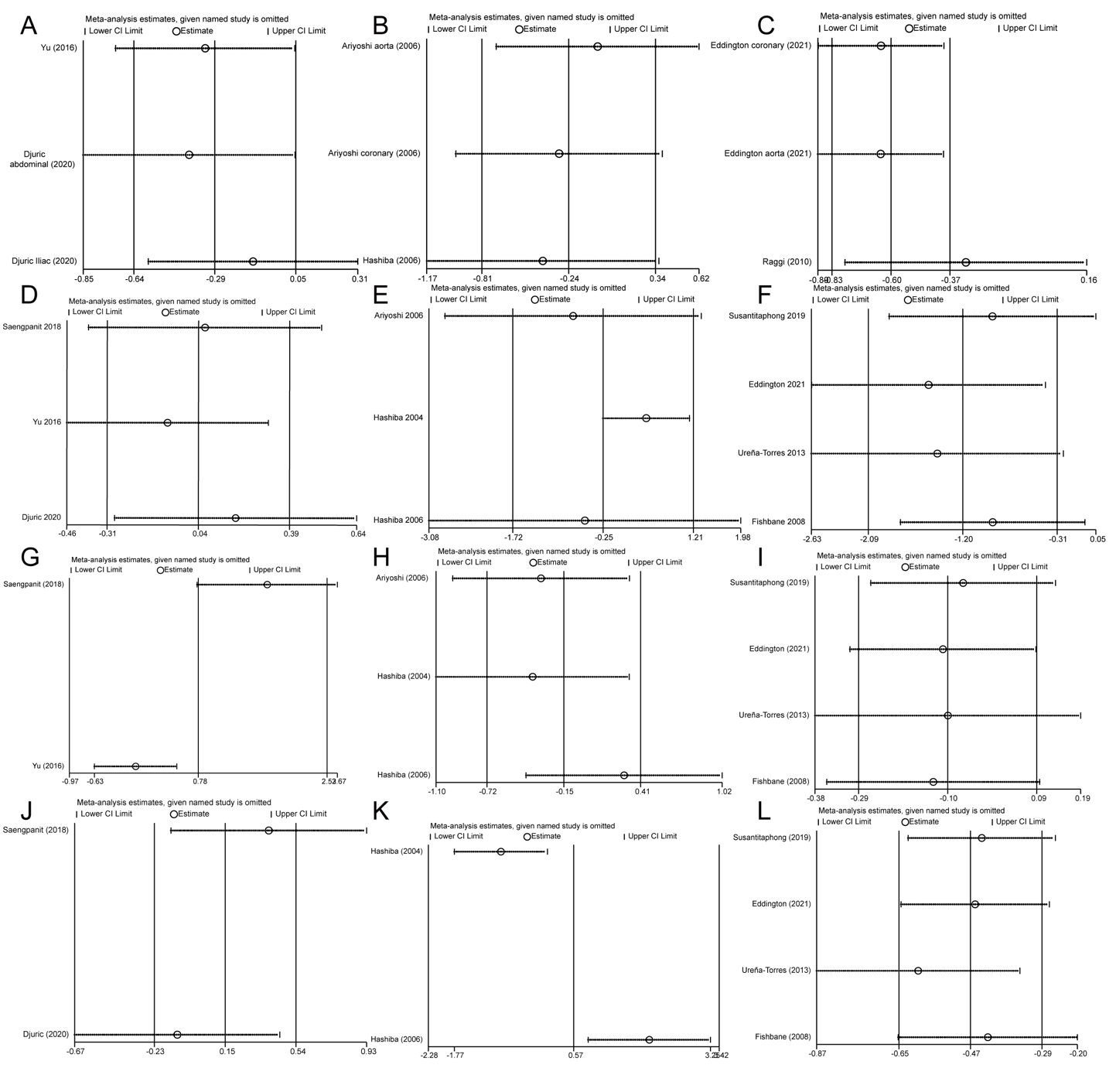


**Supplement Figure 5. Sensitivity analysis for** **each indicator treated with different medications.** Sensitivity analysis for calcification score treated with (A)sodium thiosulfate, (B)bisphosphonates, (C)cinacalcet. Sensitivity analysis for serum calcium level treated with (D)sodium thiosulfate, (E)bisphosphonates, (F)cinacalcet. Sensitivity analysis for serum phosphorus level treated with (G)sodium thiosulfate, (H)bisphosphonates, (I)cinacalcet. Sensitivity analysis for serum iPTH level treated with (J)sodium thiosulfate, (K)bisphosphonates, (L)cinacalcet.


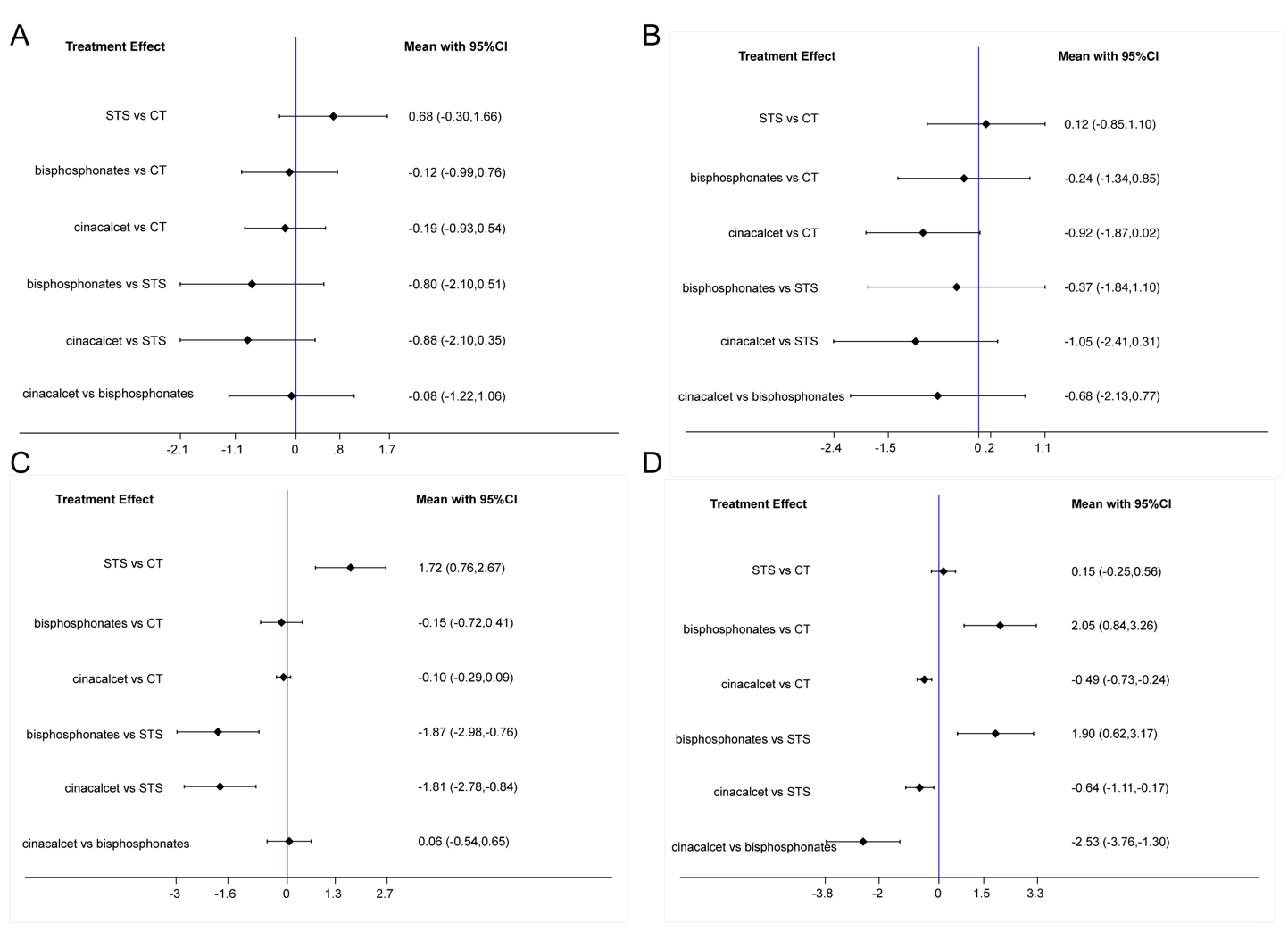


**Supplement Figure 6. network meta-analysis after sensitivity analysis for each indicator.** (A) Interval plots for calcification score. (B) Interval plots for serum calcium level. (C) Interval plots for serum phosphorus level. (D) Interval plots for serum phosphorus level.
